# Vaccine value profile for norovirus

**DOI:** 10.1016/j.vaccine.2023.03.034

**Published:** 2023-10-06

**Authors:** George Armah, Ben A. Lopman, Jan Vinjé, Miguel O’Ryan, Claudio F. Lanata, Michelle Groome, Jared Ovitt, Caroline Marshall, Elizabeth Sajewski, Mark S. Riddle

**Affiliations:** a Noguchi Memorial Institute for Medical Research, University of Ghana, Legon, Ghana; b Department of Epidemiology, Emory University Rollins School of Public Health, Atlanta, GA, USA; c Division of Viral Diseases, National Center for Immunization and Respiratory Diseases, Centers for Disease Control and Prevention, Atlanta, GA, USA; d Microbiology and Mycology Program, Faculty of Medicine, University of Chile and Instituto de Sistemas Complejos de Ingenierìa (ISCI), Santiago, Chile; e Instituto de Investigación Nutricional, Lima, Peru; f National Institute for Communicable Diseases, National Health Laboratory Services, Johannesburg, South Africa; School of Pathology, Faculty of Health Sciences, University of the Witwatersrand, Johannesburg, South Africa; g Office of Medical Research, University of Nevada, Reno School of Medicine, Reno, Nevada, USA; h World Health Organization, Geneva, Switzerland

**Keywords:** Vaccines, Policy, Global Health, Vaccine value

## Abstract

Norovirus is attributed to nearly 1 out of every 5 episodes of diarrheal disease globally and is estimated to cause approximately 200,000 deaths annually worldwide, with 70,000 or more among children in developing countries. Noroviruses remain a leading cause of sporadic disease and outbreaks of acute gastroenteritis even in industrialized settings, highlighting that improved hygiene and sanitation alone may not be fully effective in controlling norovirus. Strengths in global progress towards a Norovirus vaccine include a diverse though not deep pipeline which includes multiple approaches, including some with proven technology platforms (e.g., VLP-based HPV vaccines). However, several gaps in knowledge persist, including a fulsome mechanistic understanding of how the virus attaches to human host cells, internalizes, and induces disease.

## 1. The global public health need for a vaccine

Norovirus is attributed to nearly 1 out of every 5 episodes of diarrheal disease globally and is estimated to cause approximately 200,000 deaths annually worldwide, with 70,000 or more among children in developing countries [1]. While norovirus is ubiquitous among all populations in high-, middle-, and low- income settings, incidence is highest in young children and at an earlier age among children in low-and-middle-income countries (LMICs). In regions where rotavirus vaccine has been introduced, norovirus-associated illness are often the leading cause of medically-attended visits for acute gastroenteritis in pediatric populations, likely via replacement [2–5] An individual will experience an average of three to eight norovirus illness episodes in their lifetime, of which at least one will occur by age of 5 years. Noroviruses are transmitted by multiple routes, but person-to-person spread predominates. Noroviruses remain a leading cause of sporadic disease and outbreaks of AGE in industrialized settings [1], highlighting that improved hygiene and sanitation alone may not be fully effective in controlling norovirus. Current infectious disease priorities are largely based on the burden associated with medically attended health events. However, based on current models, the overwhelming cost of norovirus is due to productivity losses resulting from acute illness. Productivity losses tend to go unrecognized but make up 94 % of the global economic burden of norovirus [6]. From a full public health value approach, focusing only on medically attended outcomes substantially underestimates the total economic impact of infections, particularly norovirus. In summary, due to the ubiquitous nature of norovirus and the substantial health and economic impacts across high-, middle-, and low-income countries, norovirus gastroenteritis should be considered a global health problem which cannot be addressed by hygiene and infrastructure alone. Table 1 and Table 2 provide a summary of the epidemiology, potential indirect public health impact, key populations and associated delivery strategies relevant to a norovirus vaccine.

### 1.1. Current methods of surveillance, diagnosis, prevention, and treatment

#### Surveillance –

Many HICs such as Japan, the United States, Germany, and parts of China have implemented active and passive systems for monitoring norovirus infections and outbreaks [31,40–42]. However, most LMICs and regions of the world do not include assessing for norovirus at present. Although the Middle East and North Africa (MENA) region employ active and passive surveillance to monitor a number of disease pathogens, they currently do not include assessment for norovirus [43]. Similarly, in Latin America, there is no active surveillance of norovirus, and all data on disease distribution and determinants are collected through epidemiologic field studies [44,45].

#### Diagnosis –

Methods vary by country and geographic region based on available resources. In HICs, diagnosis is generally presumptive due to the appearance of one or more norovirus related symptoms such as loose stool or vomiting and occurrence during the winter months. When routine clinical laboratory diagnosis is sought, it is usually tested via realtime (RT)-PCR platforms. However, due to most cases not presenting to medical services and the lack of RT-PCR availability and testing generally, norovirus cases often go undiagnosed. This is an important problem, since most physicians and pediatricians in LMIC are unaware of norovirus as an important agent, and therefore, they do not see the need for a norovirus vaccine, which might limit its use once it becomes available (*Riddle et al, manuscript in preparation*).

#### Prevention –

Individual protection against norovirus (and other enteric viruses) occurs through routine practice of hygiene measures such as proper hand hygiene, safe food handling and preparation, surface cleaning and disinfection, and thorough laundry washing. From a population health perspective, identification and mitigation during outbreaks are important to mitigate spread with in communities.

#### Treatment –

Current standard of care for norovirus infection remains supportive, with focus on hydration and electrolyte replenishment. Antiemetics and antimotility agents may play a role in some patients [46].

### 1.2. Summary of knowledge and research gaps in epidemiology, potential indirect public health impact and economic burden

#### Epidemiology

- Data underlying the illness burden estimates in adults and children over 5 years of age are sparse and subject to important under-reporting bias, especially in LMIC. Current estimates are significantly challenged by the fact that norovirus infections and detection of outbreaks largely go unrecorded as most people do not contact health care services because of the nature of the infection.
- There is a need to genotype norovirus strains associated with more severe AGE in different parts of the world, specifically in pediatric populations, to select the best combination of antigens for an effective norovirus vaccine that could be used globally.
- Data are lacking on the potential chronic health effects due to norovirus (e.g., post-infectious irritable bowel syndrome, constipation, dyspepsia, and gastroesophageal reflux disease).
- There are significant gaps in global norovirus surveillance that impose barriers for accurate estimation of global norovirus disease burden.
- Current observations on outbreak activity reflects the bias of information attainment from settings in congregate settings, and therefore may underestimate other important outbreak activity due to norovirus.

#### Indirect public health impact

- Societal costs include direct and indirect (i.e., productivity losses due to absenteeism from work or school and mortality) costs.
- Modelling suggests that productivity losses due to absenteeism is a large driver of total disease burden.
- There is an important need to measure indirect effects of norovirus infections in LMIC in nutrition (growth velocity) and cognitive development, as seen with AGE in general.
- For HIC (and LMIC as well), the impacts on work-force absences in healthcare and education settings, hospital infection control and isolation costs, as well as impacts on immunocompromised individuals also need evaluation.

#### Economic burden

- Current estimates of the economic burden include studies that exclude vomiting-only norovirus episodes, which may represent 13 %–27 % of cases in the community [16,47], and thus likely underestimate the true cost burden of all norovirus illnesses.
- There are limited data on treatment administered outside the formal health system (e.g., local pharmacy over the counter, traditional healers, oral rehydration) but would likely be substantial in the societal and individual costs of disease.
- More reliable data on health care seeking behavior, hospitalizetion rates for norovirus, and missed productivity, especially for LMICs and older children and adults would be particularly useful.

## 2. Potential target populations and delivery strategies

### 2.1. High-income countries

While no formal target product profiles exist, from an epidemiological perspective, likely population segments for a norovirus vaccine could include all age-groups given the epidemiology of infection and disease burden associated with infection. Priority groups in HICs would likely include children, the elderly, and travelers as well as certain industries such as food-handling, health care and education systems where outbreaks are frequently known to occur and cause substantial disruptions. Of interest, Bartsch and co-workers employing modelling techniques, have suggested that the greatest potential economic and health benefits of a norovirus vaccination programme will be young children (under 5 years old) and the elderly (over 65 years old) [48]. Within HICs a norovirus vaccine could be integrated into the routine childhood vaccine schedule as well as routine adult immunization schedules. Traveler populations could be identified and immunized as part of routine pre-travel counselling.

### 2.2. Middle-income countries

As with HIC, norovirus is likely to impact all segments of the population in middle-income countries. Immunization systems are robust, and a pediatric vaccine would likely be able to be integrated into *EPI* schedules. However, many MICs have not introduced rotavirus vaccines with which a new norovirus vaccine may compete. From an adult vaccine preventable disease perspective, a recent review has captured current challenges broadly for adult vaccine introduction in middle- (and low-) income countries [49]. In the next decades, the number of adults over 65 years of age will grow to be more than the under-5 population, heavily concentrated in low- and middle-income countries. Current adult vaccine programs targeted at pneumococcal disease, influenza, and herpes zoster provide prescient examples of gaps and considerations for adult immunization programs in these countries. Lack of burden of disease in adults limits the potential public and governmental assessment of adult immunization program value. The few countries reporting adult immunization programs generally focus on high-risk groups, and a norovirus vaccine may not compete well with the other aforementioned vaccines. There is also a general lack of appropriate delivery platforms. Thus, a robust system for norovirus vaccination in adults may not be easily integrated into many MICs that could benefit from a norovirus vaccine, except for high-risk adult populations that some MICs target.

### 2.3. Low-income countries

Approximately 70 % of norovirus cases worldwide are known to occur in children between the ages of 6 and 23 months with the median age of infection in LMICs, where the burden of norovirus infection is highest, being between 6 and 16 months of age [31]. A vaccine should also provide protection before the peak of infection. Pediatric immunization could be implemented within the existing *EPI* or immunization schedule. Since the burden of it is greatest during the first year of life, immunizing early will have the greatest impact. It is estimated that a norovirus vaccine schedule completed by 6 or 12 months of age could prevent up to 85 % or 50 % of pediatric cases, respectively. The introduction of any new vaccine into routine vaccination programs in most LIC require a complex set of activities including mobilization and leverage of political will and country leadership; advocacy and communications; advanced planning for all aspects of vaccine introduction including processes, standard operating procedures, preparation of the cold chain, logistics, training, and monitoring and evaluation. Most countries in the LMIC, where the burden of disease is quite high, currently deliver vaccines against 13 vaccine preventable diseases (VPDs) at regular schedules within their immunization programme. The current *EPI* schedule is contact at birth, 6 weeks, 10 weeks, 14 weeks, 6 months, 9 months, 12 months and 18 months. Immunization could be implemented within this schedule. It should be noted that the success of a pediatric vaccine will depend very much on how it can be integrated into the current immunization programmes of countries. As stated above, issues with adult immunization programs in MIC would be the same for LIC.

While the above considerations have been broken out by socioeconomic strata, these likely have applicability across all settings. Other target population and use-case considerations across all socioeconomic strata are important to consider including situations when a novel pandemic strain might arise, utilization of the vaccine as part of an outbreak response in congregate, health care and educational settings, as well as the potential value of a norovirus vaccine in maternal vaccination programs.

## 3. Norovirus and its consideration as a public health priority by global, regional or country stakeholders

Norovirus vaccine stakeholders are thought to include global health organizations, low-income countries where mortality and morbidity are considerably high, and high-income countries in which burden of disease has been substantially defined and vaccine markets may support early introduction, as well as non-governmental philanthropic organizations (Table 3). For countries who have introduced rotavirus vaccines into their national immunization programs, norovirus would likely be a pathogen (along with shigella) for a vaccine target of interest. However, such an assessment is largely hypothetical as there is little documented evidence that defines the prioritized interest in global health intuitions and stakeholders. At the country-level there is better evidenced interest by the United States as well as China, though formal estimates of demand and vaccine uptake are lacking. Within the United States, health economic analyses have been conducted for US general population as well as the US Department of Defense.

A norovirus vaccine should have a dual market potential with pediatric populations in LMIC and HIC, as well as adult populations in HIC and MICs. Private markets in MICs may be sizeable given the burden of disease of this pathogen in all segments and potential demand for such a vaccine. However, formal market analyses are clearly needed.

## 4. Existing guidance on preferences/preferred product attributes for vaccines against norovirus

Neither the WHO nor any other global priority setting body has developed a preferred product characteristic or target product profile for a norovirus vaccine.

## 5. Vaccine development

### 5.1. Probability of technical and regulatory success (PTRS):

There are a number of features currently known about a norovirus vaccine that are favorable towards development of successful norovirus vaccine. These include a diverse though not deep pipeline which includes multiple approaches, and some with technology platforms that have proven successful for other vaccines (e.g., VLP-based HPV vaccines).(Tan 2021) In addition, there is evidence for acquisition of immunity from natural exposure [53,54]. A fairly clear development pathway exists for pediatric populations in LMIC (e.g., rotavirus vaccine development), and there is a human challenge model that could provide opportunity for de-risking, though this model has not been extensively used [55–58].

However, development of a norovirus vaccine is not without challenges. Mechanistic understanding of how the virus attaches to human host cells, internalizes and induces disease is not fully elucidated, and which genotypes to include may rely on future emerging new strains as well as the level of cross-protection against different genotypes that are not included in the vaccine formulation [59,60]. Hence, the frequency of possible reformulation of a norovirus vaccine is currently unknown. There are no good animal disease models that recapitulate human disease, though the recently developed human intestinal enteroid system may provide newer tools to better understand biology and vaccine design optimization.[61].

Table 4 outlines the current expert assessment of the PTRS for a norovirus vaccine with a standardized rating provided according to Appendix A. Table 5 provides an overview of parameters that inform scientific feasibility of developing an effective vaccine for LMIC public market use.

### 5.2. Overview of the vaccine candidates in the clinical pipeline:

**Fig. 1.**
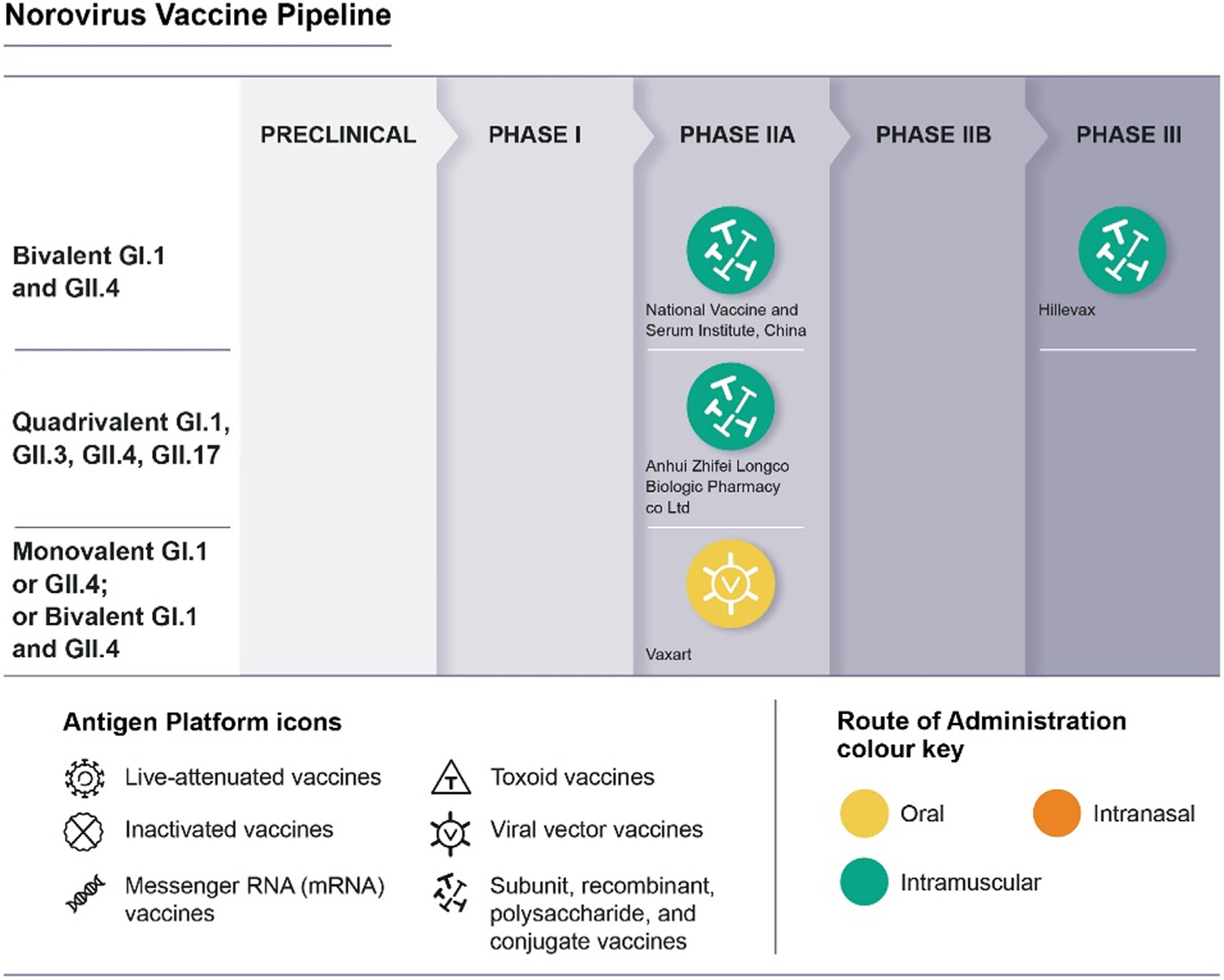
30th, 2022 (Credit: WHO).

There are currently-three VLP-based platform vaccine candidates and one adenovirus vector-based platform which have undergone clinical studies (Fig. 1 and Table 6). The most advanced candidate has produced several publications, from its early stage (Ligocyte) and later stages (Takeda) including phase I and II trials as well as formulation and dosing adjustments and active search of immune correlates of protection. This vaccine, currently under development by Hillevax, is the only candidate reporting clinical efficacy to date, and is moving forward to phase III trials in adults and children from 5 months of age. The other two VLP candidates, from Chinese manufacturers are in earlier stages, and peer-review results of the phase I trials have not yet been reported. The adenovirus vectored GI.1 vaccine using oral tablets was reported to be safe and immunogenic (using several immunologic parameters).

## 6. Health, social and economic impact of a norovirus vaccine on burden of disease and transmission

A norovirus vaccine would have a number of different target populations and thus a variety of potential health, social and economic impacts (Table 7). These populations include young children in LMICs as well as those in HICs. In addition, there are healthy adults, as well as older adults where there is substantial burden. Finally, there are also identified specific at-risk populations such as healthcare workers or food-handlers. As such, there are many potential and important impacts that a vaccine may have. The tables below provided substantial detail on what has been considered thus far. From an age-based perspective, the youngest will have the highest rates of overall healthcare utilization, as well as are considered primary drivers of transmission in communities. In HIC, more severe disease is also found in the older age groups, with a majority of deaths occurring in elderly individuals aged 65 years and older. Travelers from HIC to LMIC are at increased risk of itinerary disrupting illness which not only has individual health impacts but could also impact the local economies through reduction of purchases/expenditures during travel. Military personnel in garrison, on ships and deployed on the ground in overseas locations also have a high incidence of norovirus illness which could impact readiness and operational effectiveness. Other high-risk groups include health-care workers and food-handlers, the latter of which would be important given that norovirus accounts for a considerable amount of domestically-acquired foodborne illness in HIC.

### 6.1. Summary of knowledge and research gaps in modelling health, social and economic impact on disease burden and transmission

Gaps in modelling literature include:

- Impact of norovirus vaccination in settings where vulnerable population are at risk such as long-term care facilities.
- Impact of norovirus vaccination across the whole population considering the role of and targeting vaccination among high transmission populations (e.g., healthcare workers, food workers and handlers).
- Comparison of vaccination schedules in young children, considering maternal antibody interference and norovirus infection rates.
- Impact of vaccination in LMICs. There are some models from middle income countries but few and none in LICs.
- Models that consider the multi-strain dynamics and vaccine valency.
- Models which consider the impact of COVID-19 and changes in underlying disease dynamics of norovirus, including the proportion of susceptible individuals in different age groups, and possible disruptions to inter-epidemic cycles.

**Influential model inputs** that need further definition to inform modelling studies include:

- Care seeking behaviors and health care utilization for norovirus across diverse age groups and populations (especially in LMIC settings).
- Protection and duration of protection of norovirus immunity following vaccination and/or viral infection.
- Impact of maternal antibodies on vaccine effectiveness in young children.
  - Cost of norovirus vaccine and vaccine program implementation.
  - In children.
  - In older adults.
  - In the military.
  - Other targeted groups like HCWs.
- Vaccine uptake willingness of norovirus vaccine among priority populations.
  - Children, older adults, the military.
- Established cost-effectiveness cut off value across settings (not norovirus specific).

## 7. Policy considerations and Financing

The evidence required to support a global policy recommendation and financing from Gavi are quite similar to other acute enteric vaccines, most notably rotavirus vaccines for which there are a number of oral rotavirus vaccines already pre-qualified and under the Gavi investment. In addition, new parenteral rotavirus vaccines are currently under development also provide an example pathway for development, licensure and lessons learned from policy development and financing perspectives.

In general, the pathway for a norovirus vaccine would be quite similar to a rotavirus vaccine, although given the epidemiology of norovirus the evaluation of the full public health impact of disease extends beyond childhood given that norovirus incidence and burden of diseases is present and quantifiable across older children, adolescents, adults and the elderly. Given that the severity of illness and under 5 mortality attributable to norovirus appears less in children compared to rotavirus, the full public health burden for those age greater than 5 needs to be considered and enumerated.

Experience also indicates that country level preferences need to be considered early in the process of development. For example, recent evaluation on the hypothetical introduction of injectable or oral next generation rotavirus vaccines (NGRVs) identifies that vaccine delivery considerations were the most important preference drivers for national stakeholders, followed by improved efficacy and cost [80]. Interestingly, while national vaccine program stakeholders preferred a higher efficacy stand-alone injectable NGRV or existing oral live-attenuated rotavirus vaccines, health care providers strongly opposed an injectable rotavirus vaccine to a vaccine schedule. From both immunization program and provider stakeholder perspectives, combination vaccine approaches are much preferred compared to stand-alone vaccines and thus early considerations of combination norovirus vaccines with existing vaccines or those in advanced development (Phase III) could be considered.

Finally, considerations of Gavi financing for new vaccines is critical to garner interest by industry partners, though the potential dual market of a norovirus vaccine may provide non-Gavi financing from high and middle-income countries based on the potential value of preventable disease burden by vaccines.

From a non-Gavi eligible country perspective (e.g., MICs and HICs), vaccine policy and financing considerations would be largely driven by identification of substantial disease burden, demonstration of efficacy in a country or region, approval by a national regulatory authority and consideration and endorsement by a national immunization policy making body. Financing of the vaccine would likely be a blend of private and public market procurement.

Table 8 outlines a number of policy and financing considerations from an LIC perspective that apply in large part to all vaccines but have been made specific to a hypothetical norovirus vaccine as appropriate. Many considerations cannot be known at the present due to the early development process of current norovirus vaccines.

## 8. Access and implementation feasibility

Vaccines only work if they are actually used. And getting vaccine manufacturers to pursue development and licensure of a vaccine requires a strong market and one that is sustainable long term. Thus, in identification of market strength and sustainability, issues of vaccine access and implementation are of critical importance to consider in prioritizing, developing and establishing policy and financing of novel vaccines such as norovirus. Pertinent to a novel norovirus vaccine, the rotavirus vaccine experience is instructive and provides perspective on what might be relevant to consider. While rotavirus vaccines have consistently been shown to save lives in many LMICs, and to be highly cost-effective or even cost-saving in many, 110 countries have introduced rotavirus vaccines into their national immunization programs, 53 have accessed support from Gavi, and 65 countries have not expressed plans to introduce rotavirus vaccines, including 8 that are eligible for Gavi support.[87]

Despite the clear evidence of mortality reduction by rotavirus vaccines, the high emphasis on infant mortality reduction by global vaccine financers and regional immunization program authorities often presents a challenge for diseases where there is ‘relatively’ more morbidity than mortality. Such is the case with rotavirus and would appear to be even more of the case with norovirus. To offset the lesser case-fatality rate of norovirus and relevant to the situation of non-rotavirus adoption among many LMICs, a framework was used to consider the favorability of access and implementation and ultimately market strength and sustainability (Appendix B). While limited vaccine candidates are currently in the pipeline (and early stage at that), we considered both a parenterally delivered VLP-based vaccine construct (Hillevax, Boston, MA USA) and an oral adenovirus vectored- VLP-based construct (Vaxart, South San Francisco, CA USA) as prototype vaccines in the access and implementation framework. For simplicity we refer to these as oral novel norovirus vaccines and injectable novel norovirus vaccines.

### Possibility of implementation within existing delivery systems

Both oral and injectable vaccines are common vaccine constructs on the market therefore theoretically implementing with existing delivery systems is possible. However, the unique product profiles and formulation characteristics of a vaccine would have important impacts on whether such a new vaccine could be integrated into existing systems. Cold storage requirements, shelf life, and package volumes would be important, but final formulations/-packaging for both constructs are not known at this time. Vaccines which do not require significant cold storage demands, have a long shelf life and are contained in environmentally friendly and space efficient packaging are optimum. Unique to a currently developed adenovirus-vectored norovirus vaccine by Vaxart, this vaccine is formulated as oral tablets (or liquid formulation for patients unable to swallow tablets) which are room temperature-stable. [88] While there are no existing systems which deliver tablet-based vaccines globally, such a formulation could be conceivably integrated and would be an alternative to the current crowded landscape of injectable vaccines.

Vaccine schedule is also an important feature to consider in this area of implementation feasibility. Current trials of available constructs are considering both single dose and two dose regimens separated by 1 or 2 months with boosters at 4 months (see vaccine section). Single dose regimens could be better accommodated in schedules, though multi-dose regimens may be required particularly in younger populations who are not already primed by natural history exposure.

Finally, potential for combinability with other licensed or near-to license vaccines is a consideration. An attractive combination would be a norovirus combined with rotavirus vaccine given the overlap in clinical condition, as well as the global epidemiology of norovirus. Oral and next generation parenteral rotavirus vaccines are currently being used or may be introduced into the near future. An alternative opportunity might also be to make a combined influenza-norovirus vaccine given the similar geographic distributions (global), strong overlap in seasonality, and the potential need to update the norovirus vaccine with prevailing genotypes as new genotypes emerge.

Thus, the possibility of implementation within existing delivery systems is seen as HIGH to VERY HIGH.

### Commercial attractiveness

In support of favorability for this factor, based on our assessment there are large target populations in HIC and LMIC, both private and public markets. Though formal market assessments are needed, it would be likely that military and traveler markets would be quick to adopt such a vaccine, as well as food handlers and those working in congregate settings where norovirus outbreaks are known to frequently occur. In addition, given that norovirus is a recurrent illness among all age-groups and the potential that the vaccine might need to be readministered as genotype epidemiology changes, demand requirements may be higher than traditional pediatric vaccines. This may be seen as a downside as well if manufacturers are required to reformulate the vaccine at some frequency with safety and efficacy studies if correlates of protection are not established.

The potential downside for the attractiveness of a norovirus vaccine is whether the vaccine would meet considerations and be prioritized under Gavi’s Vaccine Investment Strategy. If nonmortality burden of disease is considered and augmented with broader population-based burden of disease estimates, the global public health value of a norovirus vaccine may be considerable and of high priority. If the vaccine has a large global market in HIC and middle-income countries who are willing to pay, this may also increase interest given the opportunity for cost-sharing of a high-volume vaccine with tiered pricing.

Given these reasons, the commercial attractiveness for a norovirus vaccine at present is assessed as MODERATE to HIGH.

### Clarity of licensure and policy decision pathway

Rotavirus vaccines (and other acute diarrheal vaccines under development) have paved the way for clinical development, licensure and policy decision pathways. Given the attractiveness of a norovirus vaccine to HIC populations, it is highly likely that a recognized NRA would license the vaccine for their populations including pediatric age-groups. The WHO SAGE in working on rotavirus vaccines has an existing framework to consider a norovirus vaccine, though would need to be expanded to consider the full public health value of a vaccine that extends beyond children under 5 years.

Thus, we assess the clarity of licensure and policy decision pathway to be VERY HIGH for a norovirus vaccine.

### Expected financing mechanism

A norovirus vaccine would likely have fairly expansive global interest in high income countries given the documented impact of disease burden and epidemiology. Less is known about the potential interest in LMICs with competing demands for existing licensed vaccines and other novel vaccines entering the market. Given the potential dual use market for a norovirus vaccine, large volume and tiered pricing strategies could provide cost of vaccine offsets for LMICs with advanced market commitment incentives.

Therefore, the expected financing mechanism is considered LOW to MODERATE.

### Ease of uptake

A pediatric vaccine would likely be able to be integrated into an *EPI* schedule. However, depending on the timing of introduction there may be concerns about another separate injection for norovirus vaccine. Combination of the vaccine with penta- or hexavalent vaccines, or a combined diarrhea vaccine (with inactivated rotavirus or shigella) may be attractive but would require other clinical and technical development. Alternatively, an oral formulation may also increase uptake given it would not require additional injection. Adult and pediatric dose formulations could present a challenge as well. Use of norovirus in the HIC should not present a major barrier.

Thus, it is assessed that the ease of uptake is considered HIGH.

## 9. Conclusion

The consideration of norovirus as a target for vaccine development as a priority pathogen is complex. On one hand, its incidence and impact are noted in all regions of the world as well as in both younger and older age-groups, thus making the potential value of preventing disease vast. This would also appear to be a driving factor to encourage multiple stakeholders who might want to see such a vaccine developed. However, in contrast to a number of other priority pathogens for which vaccines are being developed, the economic and health burden attributed to norovirus infections are generally due to milder disease and there is often a premium placed on vaccines that prevent infections that commonly lead to death. However, if one were to look at this infection from a holistic perspective, there is a considerable global disease burden that should place this vaccine as a priority pathogen. Furthermore, norovirus is one of a number of enteric pathogens for which there are current vaccines available and underutilized (e.g., rotavirus) as well as vaccines under development that provided competition for another enteric vaccine to develop (e.g., shigella, non-Typhoid salmonella and ETEC). Given these alternative interventions that are underutilized and under development, norovirus may not present as a global public health priority pathogen. However, to solve the current problem of enteric burden of disease as well as the important long-term health manifestations that accompanies populations with high disease burden caused by multiple infections, a ‘this AND that’ is needed perspective can be taken.

There are many positive considerations regarding the state of science, and vaccine development pipeline that encourage a positive outlook on the probability of success that a safe and effective norovirus vaccine could be developed. Burden of disease data (and ongoing public health surveillance) are broadly available to provide strong data that guides vaccine need, though measures of impact on work-force productivity and the accounting of impacts of mild-to-moderate disease need focus. There are currently-four different norovirus vaccines under development, relying on proven technology (VLPs) and currently in phase 2 field efficacy trials. Furthermore, there is a controlled human infection model that could be utilized to support or accelerate (e.g., in HIC adults) vaccine licensure. For LMIC pediatric indication, there is an existing ‘playbook’ on how to develop, test and approve enteric vaccines, particularly given the robust pathways that rotavirus vaccines have been advanced through.

More work is needed to understand how a norovirus vaccine might fit in the current payer models of Gavi, as well as middle-income and higher-income public and private markets.

## Data availability

No data was used for the research described in the article.

## Supplementary Material

Supple Table 2

Suppl Table 1

## Figures and Tables

**Table 1:** Summary of epidemiology and potential indirect public health impact.

Feature	Summary and evidence
*Epidemiology*	
Reservoir	Reservoir is human. Transmission is primarily through person-to-person and an estimated 10–15 % through contaminated food (e.g., grown or harvested from contaminated water (individual, municipal, recreational), or infected by preparer/handler, water, infected people and contaminated surfaces. Despite the ability of noroviruses to infect and cause disease in a broad range of animal species, to date there is no evidence that supports the transmission of norovirus from animals to humans. A few studies report evidence of human serological exposure to bovine and canine norovirus, but cross-reactivity might explain observations [7,8].
At-risk populations	Norovirus is ubiquitous, associated with 18 % (95 % CI: 17 %–20 %) of diarrheal disease worldwide, with significant burden of disease in high-, middle-, and low-income settings. Norovirus affects individuals across all age groups; however, the highest rates have been identified among young children. For a person with an average life expectancy of 80 years, norovirus will cause illness three to eight times [9–12].
Mortality	Globally, norovirus is estimated to cause a median number of 219,000 deaths each year (95 % Uncertainty Interval [UI]: 171,000–277,000) across all ages [6] 97 % of mortality is attributed in LMIC [6]. Its case fatality has been estimated to be 0.25 per 1000 cases (95 % CI 0.03–8.99), which is higher than rotavirus disease [13].
Morbidity	Globally, norovirus is estimated to cause a median number of 699 million illnesses each year (95 % Uncertainty Interval [UI]: 489–1,086 million) across all ages [6,14–16]. Norovirus is a leading cause of medically attended pediatric acute infectious GI illness, particularly in areas where rotavirus vaccine has been introduced [2–5]. 82 % of all illnesses are in LMIC countries [6]. There are research studies that indicate an increased risk of functional gastrointestinal disorders (e.g. functional dyspepsia, irritable bowel syndrome) among HIC populations. No data exists on such post-infectious sequelae in the LMIC populations.
Geographical and seasonal distribution	Norovirus has a world-wide distribution. In the northern hemisphere, seasonal trends have well established norovirus as a wintertime phenomenon, whereas seasonal patterns for norovirus in the southern hemisphere are less established, and it remains unclear if the lack of an observed trend is due to differences in epidemiologic factors or a lack of adequate data [17].
Gender distribution	Norovirus infections demonstrate a sex-switch preponderance at puberty (Male:Female IRR = 1.10, 95 % CI 1.02–1.19 in age = 5–14 years; Male:Female IRR = 0.75, 95 % CI 0.71–0.79, age 15–59 years). Adult women have higher exposure to foodborne infection due to the frequency in which they visit hospitals and day cares, and more frequently cook and prepare meals. It is likely that women of reproductive age represent more secondary cases than men due to infection directly from their children [18,19].
Socio-economic status vulnerability(ies) (equity/wealth quintile)	In LMICs limited access to potable water, access to health care, rotavirus vaccination coverage, poor sanitation, inadequate hygiene, and food contamination represent key drivers for norovirus infections. Household density may place a disproportionate burden of norovirus infections among households where there are more than 1 person living in a single room per room. Within HICs, children living in high income households had higher norovirus antibody titers at age 3 years compared to those in lower income households [20–22].
Natural immunity	In LMICs, children < 6 months of age are less frequently infected by norovirus than infants and older adults due to antibodies passed through breastmilk and limited opportunities for person-to-person and foodborne exposure. While the data are limited and reliant on outbreaks which may not represent sporadic infections, mathematical modelling estimates suggest that the duration of norovirus gastroenteritis immunity is 4.1 (95 % CI 3.2–5.1) to 8.7 (95 % CI 6.8–11.3) years [19–23].
Pathogenic types, strains, and serotypes	Noroviruses have been classified into ten genogroups (GI-GX) and 48 genotypes, though only viruses from GI (n = 9), GII (n = 23), GIV (n = 1), GVIII (n = 1) and GIX (n = 1) are causing infections in humans. GII.4 noroviruses are associated with higher frequency and more severity in all age groups, while in children GII.3 viruses are the most frequent genotype second to GII.4 viruses. GI.1 noroviruses have been identified as important in some settings, but overall is a rare genotype. Other genotypes may be important in other settings, particularly in LMIC, where few studies have been conducted Repeat infections with norovirus are common, but repeat infections with the same genotype are rare, suggesting that genotype-specific immunity is important [24,25].
*Potential indirect impacts*	
Anti-microbial resistance (AMR) threat	There are no antiviral treatments for norovirus, though this is an area of active development (Netzler et al. 2019) Many viral infections are inappropriately treated with antibiotics. Therefore, prevention of norovirus infections with vaccination could reduce antibiotic use and the development of attendant AMR (theoretical). There is an important proportion of norovirus-positive AGE episodes where other enteropathogens are also present (mixed infections), including bacterial agents like ETEC or *Shigella sp*, where antibiotic treatment may be needed. There is a need to identify which organism is the cause of AGE in episodes where more than one enteropathogen is present, to reduce un-needed antibiotic treatments.
Epidemic and outbreak potential	Outbreaks are commonly found in settings involving semi-closed populations and are particularly common in health care settings such as long term care facilities (LTCF) as well as school settings among young children of primary school and kindergarten ages. Additional studies have indicated outbreaks on ships and military camps Impacts on hospitals operations in many countries have been described [20,26–28].
Transmission route/potential	Noroviruses are transmitted through the fecal-oral route but can also spread through fomites and airborne vomitus droplets. Noroviruses are highly contagious, and outbreaks occur from direct person-to-person contact but can also be transmitted through contaminated food or water. Typically, transmission occurs from humans to foods which can then act as vehicles of infection [26,29–35].
Acquired/herd immunity	Compelling data on acquisition of natural immunity comes from volunteer challenge studies and observational birth cohorts. Natural infection appears to confer short term protection against similar strains, lasting on the order of months to a few years. Observational data from birth cohort studies demonstrate decreasing attack rates with age as well as lower rates of infection and/or gastroenteritis following one or more infections. Empirical data on the duration of immunity is lacking; a modelling study estimated a range from about 3 to 9 years. [19,23,25,36–38]
Co-associated mortality	Estimates on the proportion of childhood death associated with noroviruses are extrapolated from proportions of norovirus identified in more severe AGE episodes requiring hospital treatment. The true role of norovirus on childhood mortality will be obtained by vaccine-introduction studies, as seen with rotavirus vaccine introduction.
*Economic burden*	
Health facility costs/out of pocket costs/ productivity costs	Globally, norovirus accounts for an estimated $4.2 billion dollars (95 % UI: $3.2–5.7 billion) in direct health systems cost and approximately $60.3 billion in societal cost each year (including productivity loses). Children < 5 years old account for the majority of societal cost at 39.8 billion per year compared to 20.4 billion in societal cost for all other age groups combined. Productivity losses associated with norovirus resulted in an annual economic burden of 56.2 billion dollars per year; half of which were due to mortality(Bartsch et al. 2016) There is a need to do cost-effectiveness analyses of norovirus vaccines including cost to society, which may have important variations by regions/countries in the world. Such cost-effectiveness analyses should consider all relevant and important outcomes comprehensively. These could include studies of different (older) age groups, vulnerable populations, and the impact of prolonging or deteriorating underlying conditions in immunocompromised individuals. Other important considerations may be the indirect effects for society including education and military readiness impacts. Traditional cost-effective studies without these indicators in countries with low infant mortality may make the vaccine too costly for DALY averted [6,39].

**Table 2:** Overview of potential target and key population(s) and associated delivery strategy (ies).

Target and key population(s)	Delivery strategy(ies)
LIC - Adult	Very difficult; adult VPD burden and surveillance systems to support vaccine programs lacking; no comprehensive WHO recommendation for adult immunization; vaccine delivery systems are not optimized.[49]
LIC - Pediatric	Key population of interest and established vaccine programs. Vaccine series would be ideally introduced prior to six months of age given epidemiology [31].
MIC – adult	Very difficult; high risk groups of HIV-infected populations and access to vaccine care is more robust. Growing elderly population and traveler markets may exist. Norovirus may be an important vaccine to introduce into these populations and thus could be a pathway [49].
MIC - Pediatric	Vaccination systems are in place for introduction in pediatric populations. Likely alignment with schedule of rotavirus vaccination.
HIC - Adult	Vaccination systems are in place for adult immunizations and high-risk target populations (elderly, hospital workers, education systems, travelers, etc).
HIC - Pediatric	Vaccination systems in place for introduction in pediatric populations.

**Table 3:** Overview of non-commercial stakeholders engaged, their interest and potential demand.

Stakeholders engaged	Summary of position/interest	Potential demand and uptake
WHO	No stated position: however, the present effort demonstrates that the WHO has interest in defining the value of a potential vaccine for global use.	No WHO-derived global demand or uptake forecasts.
China	No stated position on norovirus vaccine, though research advances in candidate vaccines and establishment of national surveillance systems suggest that this is a priority pathogen. Scientists from the Chinese Centers for Disease Control state that these efforts are essential to provide information about the evolving strain distribution and epidemiologic characteristics of norovirus outbreaks which contributes to the development of effective vaccines.	No formal demand or uptake estimates are available. Unclear the target populations that might be of interest. Given size, demand forecasts likely to be large [50].
Centers for Disease Control and Prevention (CDC)/US	Not a stated position; however, interest is clear given activity and leadership in norovirus surveillance and epidemiology in the US (and globally).	No-CDC based demand forecasts, though economic analysis and burden of disease description efforts point towards important populations of interest including pediatrics, adult and the elderly [10].
Bill & Melinda Gates Foundation	Norovirus is on a learning agenda but is not in the BMGF’s active enteric vaccine portfolio.	No demand forecast/uptake assessments described.
Department of Defense/US	No specific requirement for a norovirus vaccine articulated, however DoD has provided industry support (Ligocyte/Takeda) for norovirus vaccine development and testing.	No formal DoD vaccine demand/uptake forecast though economic analysis has described potential vaccine dose requirements and cost-effectiveness of a vaccine strategy (relative to other leading enteric pathogens) [51,52].

**Table 4:** Summary of indicators supporting an assessment of the probability of technical and regulatory success for a norovirus vaccine candidate.

Probability of success theme	Indicator	Summary	Rating
Biological Feasibility	Most advanced vaccine candidate(s)	Four candidates have undergone clinical trials with the most advance (HilleVax Bivalent GI.1/GII.4 VLP) heading into phase III trials in 2022. Other three candidates in phase I/II.	Low-Moderate
	Existence of immunity from natural exposure	Natural exposure causes asymptomatic and symptomatic infections, both of which are associated with both humoral and cellular immune responses. Cohort studies conclude that prior infection confers protection against reinfection to the same genotype and potentially to the same serogroup/different serotype; duration is unclear. Adult volunteer study results provide conflicting results, suggesting limited infection- related protection.	Moderate
	Understanding mechanisms of immunity	Recent studies focusing on host differences (HBGA phenotypes for example) may partially explain early results of lack of protective immunity conferred by a prior infection. Long term protection, mucosal/systemic immune response induction, and cross-protection against heterogeneous epitopes is not fully understood.	Low
	Likelihood of vaccine protection against the majority of pathogenic strains	Strong evidence that protection is provided against the homologous genotype. Less evidence that protection may also be against the homologous genogroup despite different genotype (although there may be differences for given serotypes). Vaccine will need to include the most common genogroup/genotypes.	Low
Product Development Feasibility	Existence of animal models to facilitate vaccine development	Mouse model and zebra-fish model are being increasingly explored. Mouse models do not recapitulate human infection and appear to have different receptor/attachment mechanisms.	Low-Moderate
	Existence of in vitro assays to facilitate vaccine development	Several antibody tests have been developed and are under evaluation. HBGA blocking antibodies may prove most helpful for establishing correlates for future studies, but this is still under evaluation. The exact host cell receptor molecule(s) necessary for attachment and invasion are not identified.	Moderate
	Ease of Clinical Development	Clinical trials following the “rotavirus clinical trials model” are likely the norm and currently being implemented for Phase III trials. Challenges will include dealing with genotype diversity and role of coinfections.	High
	Availability or need for human challenge models	Human challenge models have been important for the “proof of concept” of vaccine associated protection. Current efforts include the development of suitable genogroup/genotype candidates for human challenge. Licensure in HIC adults/travelers through use of CHIM are reasonable given recent precedence	Moderate-High

**Table 5:** Overview of parameters that inform scientific feasibility of developing an effective vaccine for LMIC public market use.

Parameter	Issues and evidence
Diagnosis/case ascertainment	Most studies related to norovirus impact have focused on case definitions (acute diarrhea characterized by increase/unformed stools, with or without vomiting or fever) together with stool testing for norovirus using mostly real-time RT-PCR. This methodology is robust with the caveat that the specific etiologic role of norovirus can be questioned due to high coinfection rates in some studies and to high asymptomatic infection in others. In young children, studies evaluating multiple pathogens frequently detect norovirus together with other viral (mainly rotavirus) or bacterial (mainly diarrheagenic E. coli) pathogens, which makes an etiologic assignment difficult. Conversely, detection of norovirus in fully asymptomatic individuals (although at a lower percent when compared to diarrhea individuals), also suggests that the sole detection of the pathogen does not imply a pathogenic role [27,62–65].
Biomarkers/Correlates of risk and/or protection	While a number of correlates of protection have been explored, they have not allowed to consistently identify a correlate suitable for vaccine trials. HBGA-blocking antibodies have showed promising results, and together with studies in Human Intestinal Enteroids represent the most relevant advances in our understanding of virus diversity and their relationship with cell adhesion and infection [66,67].
Sero-epidemiological data	Population-based studies suggest that seroepidemiology may be used as a tool to measure force of infection within a given population, and that there is genotype-specific homologous protective effect after natural exposure, with some evidence of short-term cross-genotype functional anti-body responses. Number of infections need to induce broadly protective natural antibody responses are not known, nor is the full duration of homologous and heterologous protection after natural exposure [53,54].
Clinical endpoints	Clinical endpoints for vaccine studies are just recently being proposed. Following the “rotavirus model”, moderate to severe infection based on hospitalization and/or fluid replacement requirement, and/or Vesikari/modified Vesikari scores are being considered. Detection method will likely be realtime RT-PCR and protection against the clinical outcome caused by vaccine types will likely be the primary outcome.
Controlled Human infection model (CHIM)	Volunteer studies using a controlled human infection model have been developed and are used for early vaccine evaluations. Studies aiming to identify suitable challenge strains are in progress [55,56,68,69].
Opportunity for innovative clinical trial designs	Current vaccine trials for the most advanced candidates are being evaluated in adult and child populations (see below). Trial designs are conventional and will include populations followed for up to 2 years for the primary outcome as well as reinfections and potential herd protection for family members. Human challenge studies have also been considered and could be introduced into LMIC settings given the risk of infections in adults globally.
Regulatory approach(es), including potential accelerated approval strategies	Several population segments would benefit from a norovirus vaccine and there will likely be different pathways towards licensure. From an LMIC perspective, clinical development pathways would largely follow those of current rotavirus vaccines and those under development. Primary endpoints would likely be medically attended vaccine-preventable norovirus acute gastroenteritis (e.g., moderate-severe). Study designs to demonstrate safety, efficacy and vaccine manufacturing consistency would be envisioned with a target for WHO prequalification, as well as developing world manufacturing. From a HIC perspective, the multiple segments would include all age-groups. Given the potential demand for healthy adult traveler and military populations, a development could contemplate an initial licensure in this population through use of a CHIM to demonstrate efficacy (as well as ideally identify immune correlates of protection). If a correlate of protection is confidently identified, expanded safety and immunobridging studies in older age-groups as well as young age-groups could follow. Alternatively, efficacy studies may need to be done. Given the establishment of a CHIM, licensure pathways could consider the use given the sporadic nature of disease particularly in travelers and healthy adults. Though, the traditional model of field trials in LMIC and HIC under 5 populations should be achievable given the disease incidence [1,9]
Potential for combination with other vaccines	Given the route of administration, schedule, number of doses and delivery strategy the combination of a norovirus vaccine with another respiratory and/or enteric pathogen, for example rotavirus would be attractive [1,9].
Feasibility of meeting presentation and stability requirements	Likely, yes as vaccines in development utilize similar constructs and formulation to existing vaccines in the LMICs.
Vaccine platform	Depending on the vaccine construct, a norovirus VLP vaccine would be similar to other vaccines that are manufactured by developing world manufactures.
Large scale Manufacturer capacity/interest	Uncertain. Current vaccines in the pipeline are not yet developed by large manufactures.

**Table 6:** Overview of vaccine candidates in the clinical pipeline.

Candidate	Antigen platform	Developer/manufacturer	Phase of development, population, and location	Route of administration, no. of doses, schedule
Bivalent GI.1 and GII.4	Virus-like particles Baculovirus expression system	Ligocyte followed by Takeda, now Hillevax	Clinical: Phase IIb and norovirus challenge completed in US healthy adults; advancing to phase 2b trials in children under 5 in the US, Colombia, Dominican Republic, Mexico, Panama and Peru	First delivered by intranasal route, and currently developed for intramuscular administration in two doses separated by 28–57 days [51,57,58,70].
Bivalent GI.1 and GII.4	Virus-like particles Hansenula polymorpha expression system	National Vaccine and Serum Institute, China Collaborators: Lanzhou Institute of Biological Products Co., ltd Beijing Zhong Sheng Heng Yi Pharmaceutical Technology Co., ltd. Zhengzhou University	Clinical: Phase I completed in Healthy People Aged 6 Months to 59 Years; Phase II trials in progress among Healthy People Aged 6 Months to 59 Years. China.	Developed for intramuscular administration in 2–3 doses separated by 28 days [71,72].
Quadrivalent GI.1, GII.3, GII.4, GII.17	Virus-like particles Pichia pastoris system expression system	Anhui Zhifei Longcom Biologic Pharmacy Co., ltd.	Clinical: Phase I/IIa ongoing in healthy people aged 6 weeks to 59 years (age-descending). China	Developed for intramuscular administration; doses under evaluation [73].
Monovalent GI.1 or GII.4; or Bivalent GI.1 and GII.4	Adenovirus vector-based vaccine platform including the VP1 gene; Co-expression with a doublestranded RNA adjuvant	Vaxart	Clinical: Phase I in adults completed (G1.1 vaccine); advancing to Phase II trials in the US.	Oral administration; currently evaluating number of doses [74].

**Table 7:** Overview of studies that address the potential value of a norovirus vaccine on health, social and economic impacts on disease burden and transmission.

Policy question	Assessment method/measure	Additional information specific to models	Assumptions	Outcomes/interpretation
Understand the transmission dynamics of norovirus and to predict the likely impact of vaccination.	Dynamic age-specific mathematical model of norovirus transmission and vaccination.	Model fitted to age-stratified time series case notification data available from Germany. Includes the use of a self-reporting Markov model to account for variation by age and over time in the statutory reporting of norovirus in Germany. The model uses a sequential Monte Carlo particle filter. The estimated model was then extended and applied to investigate the potential impact of a range of immunization strategies. Sensitivity analyses were performed on the mode of vaccine action and other vaccine-related parameters.	SEIR-type model with maternal immunity and vaccinated classes Asymptomatic contribute to transmission greater than 90 % coverage for children; 50 % for elderly 50 % VE for kids; 70 % for elderly Duration of protection 5 years	Routine immunization could reduce the incidence of norovirus by up to 70.5 % even when those vaccines do not provide complete protection from disease. Relative efficiency of alternative strategies targeting different age groups are dependent on the outcome. Results sensitive to assumptions on the mode of vaccine action [75].
Which age groups should be vaccinated to maximize population impact?	Deterministic, age-structured compartmental model of norovirus transmission and immunity in the U.S. population.	Model was fit to age-specific monthly norovirus-associated U.S. hospitalizations between 1996 and 2007 Simulated mass immunization ofbothpediatric and elderly populations	Assumed coverages of 90 % and 65 %, for pediatric and elderly populations respectively. Considered two mechanisms of vaccine action, resulting in lower vaccine efficacy (lVE) between 22 % and 43 % and higher VE (hVE) of 50 % Maternal immunity is short-lived and negligible Vaccine response was “take-type:”	Pediatric vaccination was predicted to avert 33 % (95 % CI: 27 %, 40 %) and 60 % (95 % CI: 49 %, 71 %) of norovirus episodes among children under five years for lVE and hVE, respectively. Vaccinating the elderly averted 17 % (95 % CI: 12 %, 20%) and 38 % (95 % CI: 34 %, 42 %) of cases in 65 + year old individuals for lVE and hVE, respectively. At a population level, pediatric vaccination was predicted to avert 18–21 times more cases and twice as many deaths per vaccine compared to vaccination of the elderly. Potential benefits are likely greater for a pediatric program, both via direct protection of vaccinated children and indirect protection of unvaccinated individuals, including adults and the elderly [76].
Is pediatric norovirus vaccination cost- effective in daycare settings?	Transmission-model-based cost-effectiveness analysis	A dynamic SEIR-like transmission model of norovirus outbreaks in daycare settings was calibrated to NORS outbreak data and adapted to include vaccination. The model incorporated detailed dynamics of pediatric transmission within daycare settings (including infection via human-to-human and fomite-to-human contacts). The economic analysis utilized an incremental cost-effectiveness ratio to compare costs and QALYS of vaccination and no vaccination (observed standard of care only, in which symptomatic children are excluded from daycare). The model did not include secondary transmission outside of daycare centers.	**Vaccination Assumptions** 90% vaccination coverage Compared 50% and 80% all-or-nothing vaccination efficacy Vaccination in addition to standard of care (exclusion of symptomatic children from childcare) Two-year modelling period **Cost Analysis Assumptions** From a societal perspective (including child medical costs and loss of productivity to parents). Two-year time horizon	**Primary Outcomes:** Due to large burden of disease, vaccinating children in daycares would likely be cost-effective. Norovirus vaccination is more costly than the standard of care but leads to more QALYs than the standard of care. Vaccination leads to modest reduction in costs to manage norovirus infections, but primarily gains QALYS (gains 253 QALYS per 10,000 children). Similar cost-effectiveness ratio to other recommender childhood vaccines. **Health Outcomes:** 50% efficacious vaccine averts 571.83 norovirus cases per 10,000 children and 0.003 norovirus-related deaths. **Costs, incremental cost-effectiveness ratio (ICER):** 50% efficacious vaccine at $200/vaccination series results in net cost increase of $178.10 per child, with an ICER of $7028/QALY Based on probabilistic sensitivity analysis, Willingness-to-pay $100,000/QALY: 94.0% likely to be cost effective Even with cost of $500 per child vaccinated and modest efficacy of 50%, vaccination is likely to be cost-effective (86.7%) at threshold of $100,000 per additional QALY ICERs most sensitive to probability of norovirus introduction within the vaccination efficacy period, days in supportive care, and quality-of-life being in supportive care [77]
Clinical impact and cost effectiveness thresholds for vaccinating children or older adults in community settings	Age-structured compartmental transmission model and cost effectiveness analysis.	**Norovirus Model:** Compartmental SEIR- like transmission model with 4 age groups: preschool-aged children (0–4 years), school-aged children (5–17 years), adults (18 – 64 years) and older adults (> 65 years) applied to the US **Cost estimates:** Compared third payer perspective, including all direct medical costs, to societal perspective included direct and indirect (productivity losses) costs. Calculated ICER use of QALYs and DALYs. **Sensitivity analysis:** Monte Carlo simulations of 2000 trials, sensitivity in terms of population size, vaccine cost, vaccine efficacy, and vaccine coverage (population of 2500 to 7500 people). **Model Calibration:** Calibrated model to US population incidence and age-specific incidence trends.	Vaccine and natural infection assumed to provide protection from symptomatic illness for one year. Assumed productivity losses for all symptomatic norovirus infections and modelled annual norovirus vaccination. ICER based on willingness-to-pay threshold of $50,000/QALY	Vaccination coverage as low as 10% can provide clinical and economic benefits **Health outcomes** with varying vaccine efficacy (25%—75%) and vaccination coverage (10%—80%): Vaccinating preschool-aged children averted 8–72% of symptomatic cases in community Vaccinating older adults averted 2% — 29% of symptomatic cases **Vaccine is cost effective at higher cost thresholds** for vaccinating preschool-aged children compared to vaccinating older adults alone (cost thresholds below using societal perspective): Vaccinating children: Low efficacy (25%) vaccine was cost effective at ≤ $445, cost saving at ≤ $370; higher efficacy (75%) vaccine was cost effective at ≤ $1600, cost saving at ≤ $1300 per vaccinated person (including vaccine, administration and associated costs) Vaccinating older adults: Low efficacy (25%) vaccine was cost effective at <$42 and cost saving at < $30, higher efficacy (75%) vaccine was cost effective at < $165 and cost saving at < $100 per vaccinated person (including vaccine, administration and associated costs) Thresholds were substantially lower from the third-party payer perspective, given that the majority of savings from averted cases are a result of reduced productivity losses [78].
Cost effectiveness of norovirus vaccine compared to other enteric vaccines for military use	Modified version of an economic model developed to evaluate the cost-effectiveness of a vaccine acquisition strategy within the Department of Defense adapted to norovirus.	Cost-effectiveness analysis for use of nor- ovirus vaccine in the military using a previously developed model that evaluated vaccines for ETEC, Campylobacter, and Shigella for the prevention of non-outbreak associated travelers’ diarrhea. One-way sensitivity analysis performed using high and low values for each input variable.	Duty days lost to acute gastroenteritis chosen as an outcome measure Incidence of norovirus-attributable illness based on systematic review of incidence of sporadic travelers’ diarrhea cases in a deployed military setting Troops vaccinated prior to every deployment based on current predeployment vaccine coverage rates Vaccine coverage: 75% Vaccine efficacy: 80% Did not consider potential for herd immunity or value of preventing domestically-acquired infection Considered purchase price of vaccine ($28.59/dose), administrative costs ($2.96/dose), and costs oftreating a vaccine-associated adverse event. One-year time horizon chosen for this model and 2013 USD costs.	Absolute cost-effectiveness of a norovirus vaccine appears to be favorable: Norovirus cost effectiveness equivalent to Shigella but not as favorable as an ETEC of Campylobacter vaccine. When adjusting case definition to account for vomiting predominant illness, the cost-effectiveness ratio per duty day lost to illness (CERDDL) for norovirus vaccine is $572, which makes it the most cost effective. The CERDDL of $1,344 compared to $776 for ETEC, $800 for Campylobacter, and $1,275 for Shigella. Norovirus vaccine adoption by the DoD could prevent 12,490 cases of gastroenteritis annually during deployment. After norovirus vaccine introduction, the number of duty days lost due to gastroenteritis predicted to drop from 4,930 to 986 per year. Annual total cost of care after norovirus vaccine introduction predicted to drop from $1,952,500 to $292,875. Total annual cost of vaccination of $6,956,775 at $60.14/vaccine administered. Sensitivity analysis: 48% of variation in the economic model accounted for by inverse relationship between duration of deployment and cost-effectiveness. Increasing duration of deployment to a year leads to norovirus vaccine cost-effectiveness approaches a cost-neutral threshold when medical treatment costs alone are considered Other most influential parameters include pathogen prevalence, incidence, coverage, probability of seeking medical treatment [52].
Cost effectiveness of norovirus vaccination in LMIC military population	Adapted economic model developed by the United States Department of Defense to evaluate cost-effectiveness of vaccine acquisition strategies utilizing a static decision tree model to compare cost-effectiveness of vaccine implementation.	Evaluated the cost-effectiveness of vaccine acquisition and implementation for norovirus, *Campylobacter,* ETEC, and *Shigella* compared with current medical management. The cost effectiveness ratio was calculated based on (1) the pathogen-specific gastroenteritis prevalence, management approaches, and treatment costs; (2) the cost of administering the vaccine in the Peruvian military population, and (3) the duty days lost to gastroenteritis averted by vaccination. Performed a one-way sensitivity analysis using high and low values for each parameter.	Cost effectiveness analysis performed in the context of the Peruvian armed forces (population of LMIC adults with high incidence of infectious gastroenteritis) for a one-year time horizon in 2019 costs. **Vaccine Assumptions:** Imperfect vaccine efficacy could protect from gastroenteritis or susceptibility to the pathogens Assumed vaccine administration before each deployment to account for waning immunity Vaccine Efficacy: 80% (minimal military parameter requirements) Vaccine associated costs: purchase price of two dose vaccine, program administration costs, adverse event treatments Vaccine cost: $13 per vaccine series (estimated vaccine costs for Peruvian military based on prices of Rotarix)	After *Shigella* vaccination, norovirus vaccination was most-cost-effective in preventing gastroenteritis-associated DDL (then ETEC and *Campylobacter*), suggesting that **norovirus military vaccination should be prioritized.** Norovirus cost effectiveness estimates compare favorably to US estimates of nor- ovirus vaccine cost effectiveness in the military, suggesting that norovirus vaccine may be cost effective in the Peruvian military. Norovirus vaccination for the Peruvian military could prevent 3870 cases of gastroenteritis and decrease norovirus- associated duty days lost from 940 to 368 days. Reduction in total annual cost of gastroenteritis care from $25.942 to $17,013 annually. Cost per duty day lost averted: $803; cost per diarrheal day averted: $199 Model outcomes were most sensitive to length and frequency of individual deployments, with longer, more frequent deployments resulting in more exposure and improving cost-effectiveness [79].
Cost-effectiveness of norovirus vaccination in children in LMICs (Peru)	Markov decision model to evaluate cost- effectiveness of a two-dose norovirus vaccine in Peru’s routine childhood immunization schedule based on two recent estimates of NV incidence (one for peri-urban region, one for jungle region).	Evaluated cost-effectiveness of a two- dose norovirus vaccine added to Peru’s routine childhood immunization schedule using a probabilistic three-box Markov model for a Peruvian birth cohort. Conducted a probabilistic sensitivity analysis using Monte Carlo simulation with 20,000 draws. Model does not include the indirect costs of norovirus vaccination nor any additional direct costs of self-treatment or home care (which may be significant), also model does not account for indirect benefits attributable to reduced shedding in the community, reduced disease transmission	Based health effects on norovirus diarrhea incidence data from community-level study in *peri*-urban area of Lima and rural jungle diarrheal surveillance site in the Amazon Basin. Assumed diarrheal incidence at 5 years old was half that at 12 – 24 months and declined linearly with time. For healthcare costs associated with norovirus, used weighted averages of urban and rural treatment costs. Assumed 85% norovirus vaccination coverage with a two-dose vaccine with 47% efficacy against diarrhea within Peru’s routine childhood immunization schedule (similar to the rotavirus vaccine). Estimated cost of $13.19 per dose (based on cost estimates for another VLP-based vaccination (HPV)) with no additional vaccination administration costs. Ran model for 60 months, cost effectiveness calculated for children < 5 years old only Costs reported in 2012 USD	**Vaccinating young children against nor- ovirus could offer economic value under the right conditions in Peru:** Potentially cost effective in scenarios with high norovirus incidence In scenarios with higher norovirus incidence (jungle setting), more favorable cost-effectiveness estimates Strongly dependent on vaccine price and efficacy based on sensitivity analysis With varied incidence rates, cost per DALY averted ranged from $15,616 to $41,512 in *peri*-urban area and from $4,483 to $14,700 in rural jungle setting. Using a willingness-to-pay threshold of the GDP per capita of Peru ($6242 in 2012), the vaccine would have to be at least 70% effective at the lowest cost of $8.50 per dose to be cost effective. If the vaccine cost $11.20 per dose, vaccine effectiveness would have to be 91% to be cost effective. The annual cost of vaccination would be 13.0 million, with $2.6 million in treatment savings, resulting in the following outcomes: Vaccination could avert 473 DALYS, over 526,000 diarrheal cases, 153,735 outpatient visits, and 414 hospitalizations between birth and the fifth year of life. The ICER for norovirus vaccination would be $21,415 per DALY averted, $19.86 per diarrhea case averted, $68.23 per outpatient visit averted, $26,298 per hospitalization averted [39]
Potential economic value of human norovirus vaccine for the United States	Markov simulation model of vaccine cost- effectiveness from societal perspective (including direct medical costs and indirect costs)	Markov model selected to determine potential impact of vaccine over time (compared to traditional decision tree model) Probabilistic sensitivity analysis (i.e., Monte Carlo simulation) for all parameters One-way sensitivity analysis for vaccine efficacy (25%, 50%, 75%), protection duration (12, 24, 48 months) and vaccine cost ($25, $50, $75)	Norovirus incidence based on community-based study of AGE from England and Wales to estimate age-specific nor- ovirus incidence. Healthcare utilization based on selfreported healthcare utilization for persons with acute diarrheal disease from the US Foodborne Diseases Active Surveillance Network. Model simulations based on 1000 individuals ages 0 to 85. Assumed 43% versus 95% vaccination coverage across every age group.	Norovirus vaccination could be cost effective, depending on price, efficacy, and duration of protection: Vaccine cost-saving for young children with minimal vaccine cost (<$25) Cost of < $1,500/case averted with highest cost ($75) vaccine with efficacy of >50% and protection of 24 months For vaccine providing 48 months protection, costs per case averted was < $700 **Clinical outcomes and costs:** Vaccination could avert 1.0–2.2 million cases (efficacy 50%, 12-month duration) Vaccine cost $400 million - $1.0 billion Vaccine savings <2.1 billion (48-month duration) Protection duration is an important driver of cost-effectiveness. Children under five are suggested as the most attractive target vaccination population, in terms of cases averted and costs, followed by older adults (>65 years) [48]

**Table 8:** Overview of expectations of evidence that are likely to be required to support a global/regional/national policy recommendation, or financing for LICs.

Parameter for policy/financing consideration	Assumptions	Guidance/reports available
Consensus building to define priority public health goals for the vaccine candidate	Need to establish norovirus as a competitive global public health priority in the landscape of all enteric infections and other priority global health pathogens. Formulate and promulgate a WHO Preferred Product Characteristics for Vaccines Against Norovirus.	WHO Technical document. From Vaccine Development to Policy: A Brief Review of WHO Vaccine-Related Activities and Advisory Processes (2017) [81]. WHO | WHO Preferred Product Characteristics (PPCs) [82].
Facilitation and acceleration of product development to achieve WHO public health goals in accordance with WHO recommend norms and standards	Improve global estimates of disease burden and better characterize the epidemiology of norovirus infection. Support further description of the spectrum of natural disease history including post-infectious consequences. Support vaccine antigen selection decisions to cover broad variation of global norovirus strains to assure adequate coverage. Develop consensus guidance about the use of LMIC human challenge models to support regulatory considerations and approvals. Support the characterization of immunological surrogates/correlates of protection. Define appropriate clinical development pathways for vaccine approval in LMIC populations (children and adults).	*WHO Technical Document. From Vaccine Development to Policy: A Brief Review of WHO Vaccine-Related Activities and Advisory Processes (2017)* [81].
Registration of product by a functional National Regulatory Authority	Given the dual use potential of a norovirus vaccine for reduction of disease morbidity and mortality in a HIC, a norovirus vaccine would likely receive either a national regulatory authority approval.	
Review of key evidence inputs by SAGE working group (WG) to inform optimal use of vaccine from public health perspective, including safety, operational issues and implementation research, and programmatic suitability, as well as the quality of the evidence, values and preferences, equity, feasibility, etc.	Detailed information on epidemiological features of norovirus disease burden globally and regionally to include age-specific mortality, morbidity, and social impact. Genotype distribution over time and the likelihood of vaccine coverage would be critical to review. Clinical considerations of the norovirus may be important including challenges in clinical management, and long-term health complications (if any). Analysis of alternatives for disease prevention and control such as emerging therapeutics, or improved strategies of ORS delivery and management of childhood diarrheal/vomiting. Vaccine and immunization characteristics would be key considerations including efficacy, herd immunity, safety, cold chain, vaccine availability, schedule, and ability to reach target populations and monitor an impact of an immunization program. Formal cost-effectiveness analyses would be beneficial, ideally at region and country level. Affordability of vaccine/cost key. Evaluation of interactions with other interventions and control strategies as well as impact of vaccine introduction on the wider health system could be considered. Social, legal, ethical considerations are entertained.	WHO Guidance for the development of evidence- based vaccination-related recommendations [83].
SAGE recommendations (from SAGE WG) to WHO are adapted into global policy published as Vaccine Position Paper	Vaccine position paper development at the World Health Organization based on SAGE recommendation is a complex, rigorous, multifaceted process involving many stakeholders and occurring over roughly a two- year timeline. SAGE accepts or modifies the proposed WG recommendations or states the need for revisiting steps in the process. In the latter case, the issue is revisited at a later SAGE meeting. SAGE decisions are reached by consensus as opposed to using a voting mechanism, thus promoting in-depth discussion of the evidence and careful weighing of benefits and harms. SAGE is the arbiter with respect to the recommendations included in the position paper and is independent of WHO. Initial draft is subject to an iterative process with multiple stakeholder reviews (both internal and external) and revisions with final publication in the Weekly Epidemiological Record.	WHO Supplement to WHO Vaccine Position Papers. Guideline Development Group [83].
Concurrent with or following SAGE recommendation for widespread use, companies can submit their dossier for WHO prequalification	While HIC countries are likely to develop a norovirus vaccine, vaccine supply will likely require production from a non-HIC country vaccine manufacturer. To be eligible for prequalification, the NRA of record would need to meet certain requirements defined by WHO. Currently, common global suppliers of prequalified vaccines for low-income country markets include those based in India and China. Partnership with the International Federation of Pharmaceutical Manufacturers Association (IFPMA) and the Developing Country Vaccine Manufacturer Network (DCVMN) regarding norovirus vaccines may be helpful. Combination vaccine approaches with IFPMA and DCVMN rotavirus manufactures may be strategic.	FDA. WHO Vaccine Pre-qualification Program. 2018 [84,85]. WHO Guidance Documents. WHO - Prequalification of Medical Products (IVDs, Medicines, Vaccines and Immunization Devices, Vector Control) [86].
Develop a package of information to meet Gavi’s approach to prioritizing adoption of support of new vaccines	Gavi’s approach to prioritizing new vaccines for investment include the following categories would need to be addressed through consultations with in-country stakeholders, peer-reviewed literature, expert and partner input, health impact modelling and analytics developed for the VIS process. Rigorous assessment of health impact to include the impact of immunization program on child mortality, overall mortality and overall morbidity. Defining of additional impact considerations with norovirus to include epidemic potential, alignment with global/regional health priorities, herd immunity, analysis of alternatives, socio-economic inequity considerations, gender inequity, and/or disease of regional importance. Development of implementation feasibility to include capacity and supplier base, GAVI market shaping potential, ease of supply chain integration, ease of programmatic integration, vaccine efficacy and safety. Formal conduct of cost and value analysis which includes vaccine procurement cost, in-country operational cost, procurement cost per event averted.	*Gavi Vaccine Investment Strategy* [80].
