# Supplementary material for: Vaccine value profile for norovirus
Source: Vaccine. Author manuscript; Available in PMC 2023 Dec 10. (PMC10710898; doi:10.1016/j.vaccine.2023.03.034)
Supplement: Supple Table 2 [file NIHMS1947006-supplement-Supple_Table_2.pdf]

Supplementary Table 2: Framework to inform access and implementation feasibility

Themes Considered	Indicators	Very Low	Low	Moderate	High	Very High
**Access and Implementation Feasibility**	Possibility of implementation within existing delivery systems	• No possibility to leverage existing delivery systems due to a complex vaccine immunisation schedule	• Some evidence that existing delivery systems could be leveraged to deliver a vaccine	• Limited use of existing delivery systems to deliver a vaccine	• Vaccine can be delivered within existing delivery systems with amendments	• Vaccine can be delivered within existing delivery systems as is
**Access and Implementation Feasibility**	Commercial attractiveness	• Poorly defined target population<br>• Disease burden mainly in LMICs but vaccine unlikely to be supported by Gavi	• Small target population predominantly in LMIC public markets<br>• Difficulty defining target population in LMICs	• Large target population distributed predominantly in LMICs with potential Gavi support	• Well-defined target population in LMIC public markets<br>• Large target populations distributed across HIC and LMIC markets	• Large target population in HIC and LMIC, both private and public markets
**Access and Implementation Feasibility**	Clarity of licensure and policy decision pathway	• A need for novel licensure and/or policy pathway, which is currently unclear	• A need for novel licensure and/or policy pathway	• A possibility to leverage an existing licensure and policy pathway with major amendments	• A clear licensure and policy pathway with minor amendments	• A clear, highly precedented, fit for purpose licensure and policy pathway currently exists

**Access and Implementation Feasibility**	Expected financing mechanism	No interest from global funders or national procurement agencies, potential for private market	Unlikely to be of interest to global funders, requiring commitment from national procurement	Potential interest from global funders, depending on public health impact data, interest from national procurement agencies	High level of interest expressed from public financing agencies such as Gavi, PAHO RF, and from national procurement agencies	Advanced purchasing commitment from, for example Gavi, PAHO RF, or other pull mechanism(s) in place
**Access and Implementation Feasibility**	Ease of uptake	• Extensive challenges with a new vaccination touchpoint required<br>• High level of clinician judgement and clinical engagement<br>• Additional extensive barriers to uptake including lack of national commitment	• Evidence of low uptake for marketed vaccines<br>• Cultural barriers, negative patient perceptions	• New vaccination touchpoint required	•Well-defined target population with likelihood of high acceptability, but possible difficulties in infrastructure for vaccination	•Well-defined target population with likelihood of high acceptability<br>• Evidence of high uptake for marketed vaccine<br>• Lack of other significant barriers to introduce a vaccine<br>• Strong national commitment to introduce a vaccine
