# Supplementary material for: Vaccine value profile for norovirus
Source: Vaccine. Author manuscript; Available in PMC 2023 Dec 10. (PMC10710898; doi:10.1016/j.vaccine.2023.03.034)
Supplement: Suppl Table 1 [file NIHMS1947006-supplement-Suppl_Table_1.pdf]

Supplementary Table 1: Framework to inform vaccine development

Themes Considered	Indicators	Very Low	Low	Moderate	High	Very High
Biological Feasibility	Most advanced vaccine candidate(s)	• Ph1, or preclinical, or no candidates in the pipeline	• Ph2 candidate	• Ph3 candidate	• Ph3 candidate with high likelihood of licensure by a WHO-listed national regulatory authority	• Licensed vaccine by a WHO-listed national regulatory authority
Biological Feasibility	Existence of immunity from natural exposure	• No evidence that natural exposure confers immunity	• Conflicting or minimum evidence	• Some evidence and/or immunity of limited duration	• Good evidence of relatively long-lasting immunity	• Well established that natural exposure confers protects against severe disease (or indicated vaccine outcome) with durable immunity
Biological Feasibility	Understanding mechanisms of immunity	• Mechanisms of pathogen induced immunity unknown	• Very limited or conflicted understanding of pathogen-induced immunity and/or immune-enhanced disease	• Some understanding of pathogen-induced immunity and whether immune-enhanced disease exists	• Good understanding of pathogen-induced immunity, however some mechanisms remain unclear; evidence that immune-enhanced disease is unlikely	• All mechanisms of pathogen-induced immunity are well understood; robust evidence that immune-enhanced disease is rare
Biological Feasibility	Likelihood of vaccine protection against the majority of pathogenic strains	• Evidence that a vaccine would not protect against majority of pathogenic strains, or gap in evidence of that a vaccine would protect against	• Limited evidence that a vaccine would protect against majority of pathogenic strains	• Some evidence that a vaccine would protect against majority of pathogenic strains	• Strong evidence that a vaccine would protect against majority of pathogenic strains	• No known strain variation that is relevant to a vaccine OR evidence that a vaccine will protect against all known strains OR new strain can be rapidly developed

		majority of pathogenic strains				
**Product Development Feasibility**	Existence of animal models to facilitate vaccine development	• Animal models do not exist and no progress has been made to identify them	• Animal models do not exist but some progress has been made to identify them	• Animal models are identified but their utility have not been confirmed	• Animal models are identified and used but the mechanisms of immunity are unclear	• Animal models are well defined and used and mechanisms of immunity are well understood OR animal models are not required for vaccine development
**Product Development Feasibility**	Existence of in vitro assays to facilitate vaccine development	• In vitro assays do not exist and no progress has been made to develop them	• In vitro assays do not exist but some progress has been made to develop them	• In vitro assays are developed but their utility has not been established or the assays have not been analytically qualified	• In vitro assays are analytically qualified and used but their fit for purpose as a relevant biomarker for decision-making or licensure has not been established	• In vitro assays are qualified/validate and are fit-for-purpose for decision-making or licensure

**Product Development Feasibility**	Ease of Clinical Development	• A complex trial design (no correlate) and a significant investment in clinical sites/infrastructure needed for testing a vaccine OR low disease incidence a significant impediment to efficacy trial feasibility	• A complex trial design needed (no correlate) that may require investment in clinical sites/infrastructure OR low disease incidence requires a large and/or long efficacy trial	• A standard trial design that may require investment in clinical sites/infrastructure	• Common trial design, potential correlate and a possibility to leverage existing trial infrastructure	• Common trial design, established correlate and a possibility to leverage existing trial infrastructure AND high disease incidence allows for an efficacy trial
**Product Development Feasibility**	Availability or need for human challenge models	• Human challenge models OR diagnostic tools are important for vaccine development but are not developed	• Human challenge models OR diagnostic tools are important for vaccine development and some progress has been made to develop them	• Human challenge models OR diagnostic tools are important for vaccine development, are developed but not used or their use is unclear	• Human challenge models OR diagnostic tools would facilitate vaccine development and are developed but their use is limited	• Human challenge models OR diagnostic tools are important for vaccine development, are developed and widely used and accepted, OR no human challenge model is required
